# Variability in objective and subjective measures affects baseline values in studies of patients with COPD

**DOI:** 10.1371/journal.pone.0184606

**Published:** 2017-09-21

**Authors:** Wayne H. Anderson, Jae Wook Ha, David J. Couper, Wanda K. O’Neal, R. Graham Barr, Eugene R. Bleecker, Elizabeth E. Carretta, Christopher B. Cooper, Claire M. Doerschuk, M Bradley Drummond, MeiLan K. Han, Nadia N. Hansel, Victor Kim, Eric C. Kleerup, Fernando J. Martinez, Stephen I. Rennard, Donald Tashkin, Prescott G. Woodruff, Robert Paine, Jeffrey L. Curtis, Richard E. Kanner, for the SPIROMICS Research Group

**Affiliations:** 1 Pulmonary and Critical Care Medicine, Department of Medicine, Marsico Lung Institute, University of North Carolina at Chapel Hill, Chapel Hill, North Carolina United States of America; 2 Department of Biostatistics, University of North Carolina at Chapel Hill, Chapel Hill, North Carolina, United States of America; 3 Marsico Lung Institute/Cystic Fibrosis Research Center, Department of Medicine, University of North Carolina at Chapel Hill, Chapel Hill, North Carolina United States of America; 4 Department of Medicine, Columbia University Medical Center, New York, New York, United States of America; 5 Center for Genomics and Personalized Medicine Research, Wake Forest School of Medicine, Winston-Salem, North Carolina, United States of America; 6 Collaborative Studies Coordinating Center, Department of Biostatistics, University of North Carolina at Chapel Hill, Chapel Hill, North Carolina, United States of America; 7 David Geffen School of Medicine, University of California, Los Angeles, California, United States of America; 8 Division of Pulmonary and Critical Care Medicine, University of Michigan Health System, Ann Arbor, Michigan, United States of America; 9 Division of Pulmonary and Critical Care Medicine, Johns Hopkins University School of Medicine, Baltimore, Maryland, United States of America; 10 Department of Thoracic Medicine and Surgery, Temple University School of Medicine, Philadelphia, Pennsylvania, United States of America; 11 Division of Pulmonary and Critical Care Medicine, Department of Medicine, David Geffen School of Medicine, University of California Los Angeles, Los Angeles, California, United States of America; 12 Department of Medicine, Weill Cornell Medical College, New York-Presbyterian Hospital/Weill Cornell Medical Center, New York, New York, United States of America; 13 Division of Pulmonary and Critical Care Medicine, University of Nebraska, Omaha, Nebraska, United States of America; 14 Division of Pulmonary, Critical Care, Sleep and Allergy, Department of Medicine and Cardiovascular Research Institute, University of California San Francisco, School of Medicine, San Francisco, California, United States of America; 15 Department of Internal Medicine, Division of Pulmonary and Critical Care Medicine and Department of Veterans Affairs Medical Center, University of Utah, Salt Lake City, Utah, United States of America; 16 Division of Pulmonary and Critical Care Medicine, University of Michigan Health System, Ann Arbor, Michigan; VA Ann Arbor Healthcare System, Ann Arbor, Michigan, United States of America; Istituti Clinici Scientifici Maugeri, ITALY

## Abstract

**Rationale:**

Understanding the reliability and repeatability of clinical measurements used in the diagnosis, treatment and monitoring of disease progression is of critical importance across all disciplines of clinical practice and in clinical trials to assess therapeutic efficacy and safety.

**Objectives:**

Our goal is to understand normal variability for assessing true changes in health status and to more accurately utilize this data to differentiate disease characteristics and outcomes.

**Methods:**

Our study is the first study designed entirely to establish the repeatability of a large number of instruments utilized for the clinical assessment of COPD in the same subjects over the same period. We utilized SPIROMICS participants (n = 98) that returned to their clinical center within 6 weeks of their baseline visit to repeat complete baseline assessments. Demographics, spirometry, questionnaires, complete blood cell counts (CBC), medical history, and emphysema status by computerized tomography (CT) imaging were obtained.

**Results:**

Pulmonary function tests (PFTs) were highly repeatable (ICC’s >0.9) but the 6 minute walk (6MW) was less so (ICC = 0.79). Among questionnaires, the Saint George’s Respiratory Questionnaire (SGRQ) was most repeatable. Self-reported clinical features, such as exacerbation history, and features of chronic bronchitis, often produced kappa values <0.6. Reported age at starting smoking and average number of cigarettes smoked were modestly repeatable (kappa = 0.76 and 0.79). Complete blood counts (CBC) variables produced intraclass correlation coefficients (ICC) values between 0.6 and 0.8.

**Conclusions:**

PFTs were highly repeatable, while subjective measures and subject recall were more variable. Analyses using features with poor repeatability could lead to misclassification and outcome errors. Hence, care should be taken when interpreting change in clinical features based on measures with low repeatability. Efforts to improve repeatability of key clinical features such as exacerbation history and chronic bronchitis are warranted.

## Introduction

The Subpopulations and Intermediate Outcome Measures in COPD Study (SPIROMICS) is an observational study of 2,981 participants including: healthy never-smokers, ever-smokers (> 20 pack/years) with preserved PFTs, and individuals with COPD classified as mild-moderate or severe by PFTs designed to aid in the future development of therapies for COPD [1]. The Repeatability Substudy embedded in SPIROMICS consisted of 98 participants who volunteered to return within 2–6 weeks to repeat their baseline visit. We designed this Substudy to quantify baseline, within-person variation, including measurement errors. The 2–6 week window of the SPIROMICS Repeatability Substudy is considered short enough to avoid changes due to disease progression, yet long enough to minimize any learning effect from the initial visit. Assessing the severity of COPD utilizes a number of objective (PFTs, 6-min walk distance), subjective [modified medical research council (mMRC) dyspnea, COPD assessment test (CAT)] and patient recall (number of exacerbations) measures utilized in determining disease progression, risk of exacerbations and treatment effects. Consistency of clinical assessments during periods of disease stability is critical to interpretation, but often goes unreported. This report adds to recent reports on functional tests in COPD [2] by describing the repeatability of the selected assessments and their potential impact on assessments of COPD used in classifying severity, stability and progression. Some of the results of this study have been previously reported in the form of an abstract [3].

## Methods

SPIROMICS is a prospective cohort study that enrolled 2981 participants at 11 clinical sites. Extensive coordinator training, monitoring and follow-up was provided to assure consistency across all sites. All subjects were age 40 to 80 years and willing to undergo the extensive study procedures. Exclusion criteria included BMI >40 kg/m^2^, cognitive dysfunction and other lung disease or clinically significant cardiovascular disease that would limit the interpretability of the measures. A history of asthma was exclusionary only for never-smokers. The full SPIROMICS study design and inclusion and exclusion criteria have been reported [1], and the Repeatability dataset is available upon request (www.spiromics.org).

The SPIROMICS Repeatability Substudy comprised 98 subjects from the largest clinical sites, who repeated their entire baseline evaluation 2–6 weeks after their initial visit. Subjects experiencing an exacerbation between the baseline and repeat study visits were excluded from this analysis. We separated the evaluations into A) objective measures [PFTs, six minute walk distance and CBC], B) measures dependent on patient recall [medical history including previous lung disease diagnosis, smoking history and exacerbations] and C) subjective evaluations [CAT[4], SGRQ [5], Functional Assessment of Chronic Illness Therapy-Fatigue [6] (FACIT-F) Score, Pittsburg Sleep Quality Index [7] (PSQ), and Medical Outcomes SF-12 [8]]. PFTs were conducted pre- and post- bronchodilation (BD) (4 puffs of Albuterol sulfate HFA plus 4 puffs of Ipratropium bromide HFA). Withholding bronchodilators was not required; time from last administration was recorded. We evaluated the repeatability of a diagnosis of chronic bronchitis (CB) using: 1) patient recall of being diagnosed with CB by a health care professional; or 2) the classic definition of chronic cough and sputum production for at least 3 months/year for the last two consecutive years[9]; and 3) from the SGRQ questions regarding cough and phlegm (cough and phlegm production several days a week or almost every day)[10]. Emphysema was assessed by 1) recall of a diagnosis by a health care professional and 2) diagnosed from volumetric multidetector-row computed tomography (MDCT) of the lungs performed at full inspiration, using an emphysema index (EI) of percent voxels in the lung field < -950 HU. GOLD staging was calculated as reported [11]. Questionnaires were administered by a study coordinator in person, and answered solely by the participant. All blood samples for CBC differentials were prepared according to a standard SPIROMICS protocol and analyzed locally at each University Laboratory. All subjects provided written informed consent, and the study was approved by the IRB at each participating site (See S1 Study Information for additional details).

### Statistical analysis

To provide reliability measurements, we calculated intraclass correlation coefficients (ICC) and kappa statistics for quantitative and qualitative traits (MRC breathlessness scale and current smoking status), respectively. When a value for a trait was missing for the baseline or repeat visits, the subject was dropped from the analysis for that trait. Bland-Altman plots were used to visualize potential changes in reliability across the spectrum of quantitative traits. In some cases, reliability measures were calculated for the entire Substudy cohort and for a subset diagnosed with COPD based upon baseline PFTs.

## Results

### Demographics

The Substudy subjects were predominately white males (except for never-smokers) and non-obese. The two COPD groups were significantly older than those without airways obstruction (p = 0.0006) (Table 1). There were no significant differences between groups in BMI, reported pack-years smoking (excluding never-smokers) or time between visits (28.9 ± 9.4 days, mean ± SD); among the ever-smoker groups, fewer participants with COPD reported smoking currently. Pulmonary function differences reflect SPIROMICS enrollment groups. Bronchodilator response, as a percent change, increased with increased disease severity. However, the absolute change in forced expiratory volume in one second (FEV_1_) was not significantly different between the strata with absolute changes in FEV_1_ of 172.0 ±233.4, 121.9 ± 150.0, 215.2 ± 148.0 and 216.4 ± 167.2 for strata 1–4 respectively (p ≥ 0.314). The increasing FEV_1_ reversibility for stratum 3 and 4 as percent change results from the decreasing FEV_1_ as disease progresses [12].

**Table 1:** Demographics, PFTs and six-minute walk.

	Stratum 1	Stratum 2	Stratum 3	Stratum 4
	Never Smokers	Non-Diseased Smokers	Mild-Moderate COPD	Severe COPD
N	11	18	38	31
Age	55.4 ± 6.4	56.2 ± 8.4	64.7 ± 8.3	63.3 ± 8.5
BMI	26.9 ± 4.5	29.5 ± 5.9	27.7 ± 5.1	26.6 ± 5.9
Gender, % Male	36	61	76	61
Current Smokers %	0	72	54	30
Pack Years	0	41.2 ± 19.4	47.3 ± 19.6	47.5 ± 19.0
Race, %				
White	64	56	71	81
Black	18	28	21	13
Hispanic	18	5	3	6
Asian	0	6	0	0
American Indian	0	6	3	0
FVC L (% Predicted)	4.19 ± 1.30 (106.2)	3.87 ± 0.75 (97.5)	3.77 ± 1.0 (94.9)	2.92 ± 1.09 (72.7)
FEV_1_ L (% Predicted)	3.42 ± 1.04 (110.4)	2.99 ± 0.63 (97.7)	2.27 ± 0.71 (75.9)	1.14 ± 0.48 (38.1)
FEF_25-75%_ L/sec (% Predicted)	3.75 ± 1.16 (130.1)	2.84 ± 0.96 (101.0)	1.16 ± 0.56 (46.2)	0.46 ± 0.37 (18.0)
FEV_1_ /FVC (% Predicted)	103.6	100	79.4	52.9
Bronchodilator Response (% change)^+^				
FEV_1_ (n)	5.5 ± 9.9 (10)	4.8 ± 5.8 (16)	12.6 ± 12.7 (25)	24.8 ± 24.2 (11)
FVC (n)	0.06 ± 6.0 (10)	0.81 ± 3.8 (16)	11.1 ± 10.3 (25)	18.0 ± 17.7 (11)
6-Min Walk Distance (M)	486.3 ± 46.6	426.5 ± 86.8	403.3 ± 111.3	329.3 ± 135.4

Values are means ± SD; n = number of subjects used in the analysis. Stratum 1 = Non-Smokers (FVC > LLN and FEV1/FVC >0.7); Stratum 2 = Non-Diseased Smokers (FVC > LLN and FEV1/FVC >0.7); Stratum 3 = COPD FEV1 >50% and FEV1/FVC <0.7; Stratum 4 = COPD FEV1 <50% and FEV1/FVC <0.7. Strata 2–4 are current or former smokers with >20 pack year smoking history.

^+^ Analysis was restricted to subjects who had not used any long-acting bronchodilator in the past 48 hours or a LABA within the past 24 hours, Tiotropium within the past 48 hours, a SABA within 6 hours or ipratropium within the last 8 hours of the baseline visit.

We compared demographics (age, BMI, height, weight, pack-years) and post-bronchodilator PFTs by groups within the repeatability population at baseline (n = 98) to the total SPIROMICS cohort (n = 2852; Repeatability Substudy subjects removed). There were no significant differences in pack-years or any of the demographic parameters, with the exception of age in Non-Diseased Smokers (p = 0.05) for whom mean ages were 56 vs. 60 years for the Repeatability Substudy and the overall cohort respectively. There was no difference between the Substudy and entire cohort in FEV_1_ percent predicted.

### Objective measures

**Fig 1:**
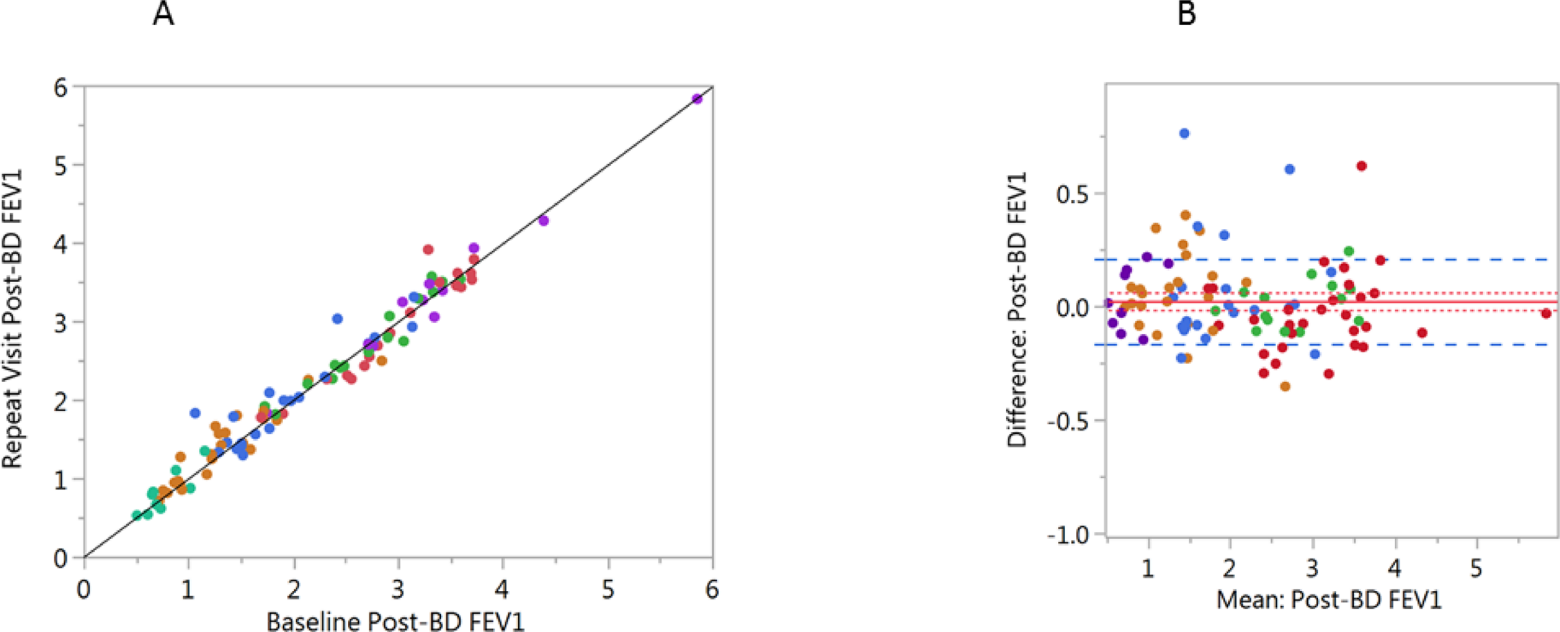
Scatterplot of baseline vs. repeat visit FEV_1_. Subjects are color coded by GOLD stratification (using PFT values only). GOLD 0 = red, GOLD 1 = green, GOLD 2 = Blue, GOLD 3 = orange and GOLD 4 = Purple. The solid black line is drawn as a line of identity to visualize differences between baseline and the repeat visit. **A)** Correlation between the baseline and repeat visit FEV_1_. (r = 0.983, p<0.0001; n = 98). **B)** Bland-Altman plot of baseline mean post-bronchodilator FEV_1_ by the difference between visits. The solid red line = the mean difference, the dotted red line is ± 1 SE and the dashed blue line is ± 1 SD.

PFTs were highly repeatable. A scatter plot and Bland-Altman plot for baseline and repeat visit post-bronchodilator FEV_1_ are shown in **Fig 1**. Data for post-bronchodilator FVC, FEV_1_/FVC and IC are presented in (**S1 Fig**). Paired differences (mean± SD) between visits were very small for post-bronchodilator FVC (0.04±0.34L), FEV_1_ (0.02±0.19 L/sec), FEV_1_/FVC (-0.001±0.04%), and IC (0.02±0.35 L). There was no apparent effect of severity on repeatability of these measures.

To reduce any potential impact of control subjects on the variability of PFTs, we calculated ICCs and the mean ± SD values utilizing only COPD subjects with an FEV_1_/FVC ratio <0.7 (**Table 2**). ICCs ranged from 0.81–0.97 for all measures, lowest for FVC and FEV_1_ bronchodilator response. The mean value of the expiratory PFT measures on the repeat visit was slightly larger than the baseline values. The ICCs for PFTs by GOLD stage remained >0.92 for GOLD 1–3 but decreased to 0.82 for GOLD 4 subjects (n = 9) (data not shown). Repeatability (ICC) of absolute post- to pre- FEV_1_ bronchodilator response for COPD subjects was 0.87 and FVC was 0.81.

**Table 2:** Baseline and repeat visit intraclass correlation coefficients for objective measures.

Clinical Measure	Baseline	Repeat Visit	ICC* COPD^+^
Pre-Bronchodilator (Mean ± SD)			
FVC (l)	3.09 ± 1.10	3.17 ± 1.10	0.94
FEV_1_ (l)	1.57 ± 0.83	1.61 ± 0.81	0.97
FEF_25-75_ (l/sec)	0.73 ± 0.54	0.74 ± 0.56	0.91
PEFR (l/sec)	4.81 ± 2.24	4.95 ± 2.21	0.96
FEV_1_/FVC	0.49 ± 0.14	0.50 ± 0.14	0.93
SVC (l)	3.19 ± 1.15	3.29 ± 1.11	0.95
IC (l)	2.44 ± 0.85	2.51 ± 0.85	0.94
Post-Bronchodilator (Mean ± SD)			
FVC (l)	3.39 ± 1.12	3.46 ± 1.08	0.95
FEV_1_ (l)	1.76 ± 0.84	1.81 ± 0.82	0.97
FEF_25-75_ (l/sec)	0.84 ± 0.59	0.85 ± 0.61	0.90
PEF (l/sec)	5.25 ± 2.31	5.39 ±2.28	0.96
SVC (l)	3.50 ± 1.17	3.59 ± 1.23	0.91
FEV_1_/FVC	0.51 ± 0.14	0.51 ± 0.14	0.96
IC (l)	2.65 ± 0.85	2.67 ± 0.87	0.93
Bronchodilator Response (%)^++^			
FEV_1_	16.3 ± 17.6	16.1 ± 15.2	0.87
FVC	13.2 ± 13.1	11.4 ± 12.0	0.81
Six minute Walk Distance	372.10 ±126.54	394.50 ± 107.91	0.79

Means ± SD

*ICC = Intraclass Correlation

+ COPD = Strata 3&4 only

^++^ Bronchodilator Response calculated as [(post-BD_-_Pre-BD) / Pre-BD FEV_1_] x 100. Analysis was restricted to subjects who had not used any long-acting bronchodilator in the past 48 hours or a LABA within the past 24 hours, Tiotropium within the past 48 hours, or a SABA within 6 hours or ipratropium within the last 8 hours of the baseline and repeat visits (n = 36).

**Fig 2:**
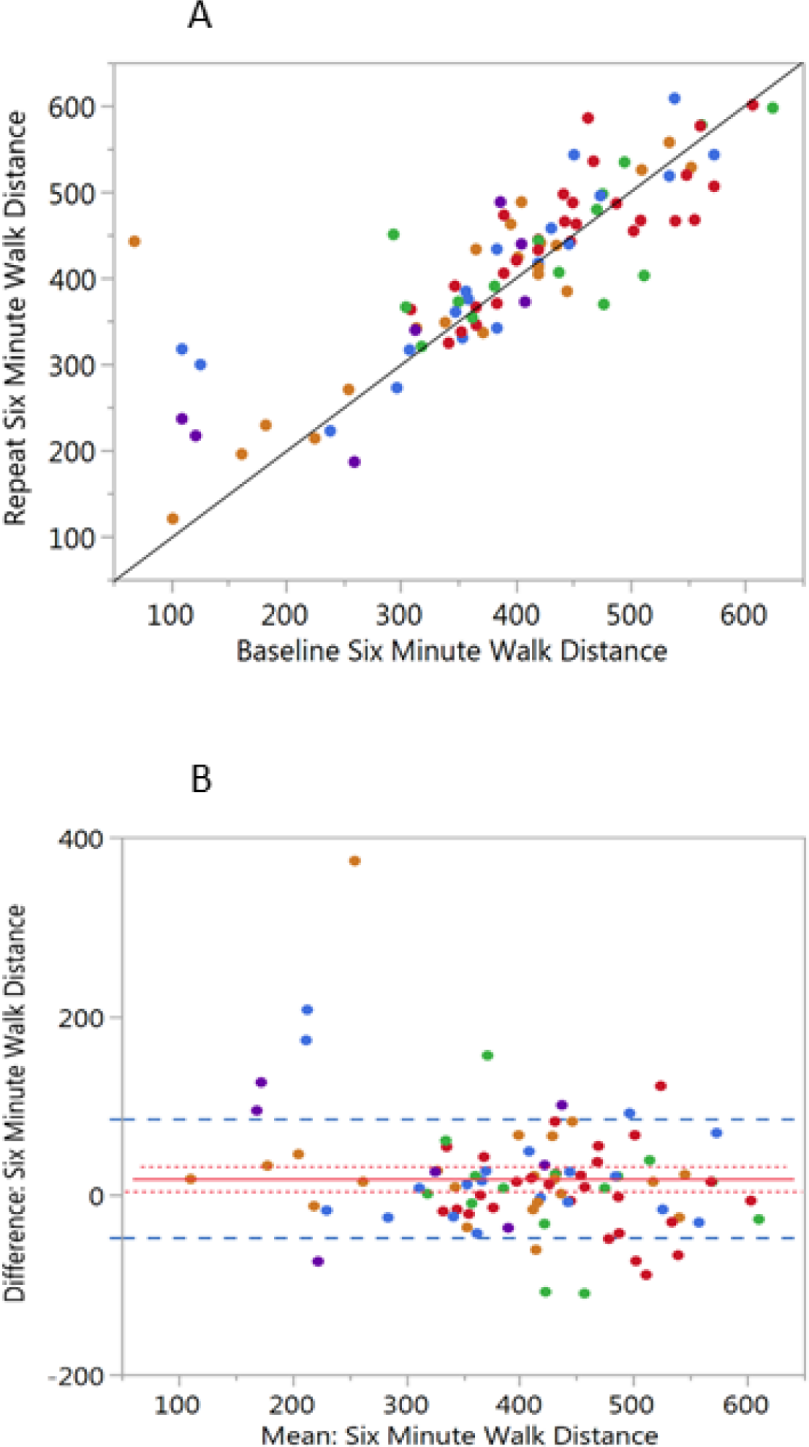
Six minute walk distance. The subjects are color coded by GOLD classification as in Fig 1A) Scatterplot of the six-minute walk distance at the baseline and repeat visits (r = 0.829, p<0.0001; n = 92). The solid black line is drawn as a line of identity to visualize differences between the baseline and the repeat visits. B) Bland-Altman plot of the mean distance for each subject (baseline and repeat) by the difference between visits. The solid red line = the mean difference, the dotted red line is ± 1 SE and the Dashed blue line is ± 1 SD.

The 6-minute walk distance (**Fig 2**) had lower repeatability (ICC = 0.79) compared to PFT measures, with a mean increased distance of 18.6 meters at the repeat visit, which is below the minimal clinically important difference (MCID) of 26 meters[13]. Subjects with the shorter walk distances were more variable between visits. Sixteen subjects (17.8%) had a decline greater than 26 meters and twenty-eight subjects (31.1%) had an increase of greater than 26 meters at the repeat visit. The mean MRC dyspnea score slightly improved from a mean of 1.5 to 1.3, consistent with the modest increase in 6-minute walk distance. Forty-one subjects (59%) had no change in dyspnea, 9 subjects (13%) increased their dyspnea score by greater than 1 and 19 subjects (28%) reported decreased dyspnea of at least 1 grade. Total white blood cell count ICC was 0.76 for all subjects and 0.72 for subjects with COPD supporting the assessment of short-term stability of the subjects between visits.

### Measures dependent on patient recall

The presence of CT defined emphysema produced highly reproducible results between visits (Kappa = 0.91) (not shown). The repeatability of self-reported physician diagnosis of emphysema was Kappa = 0.71. Patient recall of physician-diagnosed CB was similar between visits (Kappa = 0.78), but higher than that of CB determined at baseline by the classic definition (Kappa = 0.61). At baseline, 20 subjects (25%) met the classic CB definition, whereas SGRQ identified 41 subjects (48%); agreement between these definitions of CB was relatively poor (Kappa = 0.35). Recall of an asthma diagnosis by a health care professional at baseline and the repeat visit resulted in kappa = 0.57 and Kappa = 0.40 for asthma diagnosed as a child by a health care professional. (**Table 3**)

**Table 3:** Measures dependent of patient recall: Disease diagnosis and exacerbations.

Variable Name		Kappa Statistics		
	N	Kappa	95% CI	
Emphysema HCP Diagnosed	88	0.71	0.55	0.87
CB HCP Diagnosed*	87	0.78	0.61	0.95
CB Classic definition^+^	95	0.61	0.42	0.80
CB SGRQ**	82	0.61	0.44	0.78
Asthma HCP Diagnosed^##^	85	0.57	0.36	0.78
Asthma Child Diagnosis	84	0.40	0.10	0.69
Exacerbations in prior 12 months	68	0.42	0.23	0.61
Exacerbations Treated with Any Medications	68	0.45	0.23	0.66
Exacerbations Treated with Corticosteroids	68	0.54	0.31	0.77
Exacerbations Treated with Antibiotics	68	0.58	0.36	0.79
Exacerbations Requiring ED/Hospitalization	68	0.57	0.33	0.82

* CB–Chronic

Bronchitis Diagnosed by a Health Care Provider (HCP)

**+** Chronic mucus production and cough for at least 3 months/year for 2 successive years.

** Chronic bronchitis defined as cough and phlegm production several days a week or almost every day from the SGRQ.

**##** Asthma diagnosed as a child by a HCP

Exacerbation frequency is a commonly assessed parameter in COPD. We examined several “definitions” of reported exacerbations including total number of exacerbations of any severity, exacerbations requiring an emergency department (ED) visit or hospitalization, and those treated with any medication (corticosteroids or antibiotics). The repeatability of exacerbation recall was modest, but fairly consistent across exacerbation definitions (**Table 3)**. Repeatability increased if the recall of an exacerbation was associated with use of a specific medication such as a corticosteroid or antibiotic. For total exacerbations recall, 48 subjects (71%) remained unchanged, with 59 subjects (87%) unchanged in recall of ED/hospitalization exacerbations.

### Subjective measures

Subject recall of the age started smoking, average cigarettes smoked per day over the lifetime of smoking and calculated pack-years were evaluated. The baseline and repeat visit values for age started smoking were 16.4 ± 4.5 and 16.0 ± 3.5 respectively (ICC = 0.76), average cigarettes per day 25.4 ± 9.1 and 24.5 ± 9.9 (ICC = 0.79) and calculated pack-years smoked 47.4 ± 19.2 and 49.4 ± 21.6 (ICC = 0.84).

Questionnaires commonly used in COPD showed wide disparity in repeatability (Table 4). Performance ranged from SGRQ-C total score (ICC = 0.94), to MRC dyspnea (kappa = 0.42). The mean change in SGRQ-C between visits was 2.2 units; 34 subjects (55.7%) had no change above the minimum clinically important difference (MCID) of 4 units (Table 5) [14]. The two scores incorporated into the GOLD 2015 and 2017 [15] guidelines (CAT and MRC Dyspnea) appeared to have modest repeatability between visits (ICC = 0.78 and kappa = 0.42, respectively). Change between visits in CAT score ranged from +18 to -11. Among subjects with COPD, 23 of 65 (35.4%) had no change in CAT score (Table 5) above the MCID (2 units). For the MRC dyspnea score, 41 subjects (59.4%) were unchanged (Table 5).

**Table 4:** ICC values for clinical questionnaires.

Clinical Questionnaire	Baseline	Repeat Visit	ICC* COPD
MRC Dyspnea	1.5 ± 1.1	1.3 ± 1.0	Kappa 0.42^+^
Bode Index	2.5 ± 2.2	2.1 ± 1.8	0.84
COPD Assessment Test (CAT)	16.3 ± 8.5	16.2 ± 7.9	0.78
SGRQ-C Total Score	41.8 ± 21.2	40.2 ± 21.5	0.94
FACIT-F Total	111.0 ± 26.1	112.1 ± 27.3	0.91
PSQ Total Score	7.0 ± 4.2	6.9 ± 4.3	0.85
Medical Outcomes SF-12			
SF-12 Gen Health	25.0 ± 11.7	26.5 ± 12.0	0.68
SF-12 Physical Functioning	21.3 ± 12.8	22.7 ± 12.5	0.63

Data are Mean ± SD

*ICC = Intraclass Correlation, COPD subjects only

^+^ Kappa Statistic was utilized because of the categorical nature of the data

**Table 5:** Change from baseline visit.

	n	Unchanged^1^	Increased	Decreased
Total Exacerbations	68	48 (71%)	6 (9%)	14 (21%)
Exacerbations requiring ED/Hospitalization	68	59 (87%)	4 (6%)	5 (7%)
SGRQ^2^	84	27 (44%)	10 (16%)	24 (39%)
COPD Assessment Test (CAT)^2^	65	23 (35%)	21 (32%)	21 (32%)
mMRC^2^	98	41 (59%)	9 (13%)	19 (28%)
GOLD PFTs	69	53 (77%)	4 (6%)	12 (17%)
GOLD Combined-mMRC^3^	64	44 (69%)	7 (11%)	13 (20%)
GOLD Combined- CAT^3^	64	46 (72%)	9 (14%)	9 (14%)

Number of subjects (% of total). n = number changed >MCID: SGRQ = 4; CAT = 2; mMRC = 1. Increase indicates a change from A to B, B to C or C to D

Evaluation of GOLD classification determined solely by PFTs for the COPD subjects demonstrated that 53 of 69 subjects (76.8%) retained their GOLD stage at the repeat visit. (kappa = 0.75). Utilizing the GOLD symptom burden / exacerbation risk criteria [15] with MMRC, 44 of 64 (five subjects had missing mMRC scores) subjects remained unchanged (kappa = 0.54) and with the CAT assessment 46 of 64 (72%) remained the same (kappa = 0.58) (**Table 5**).

## Discussion

This formal, multi-center repeatability study, performed over an average 29-day interim in a representative subset of the entire SPIROMICS cohort, provides an unprecedented assessment of the variability of the instruments used to characterize COPD subjects. Repeatability of pulmonary function testing was strongest (ICC >0.90) and subject recall of a childhood diagnosis of asthma weakest (kappa = 0.40), consistent with a trend of greater repeatability of objective measures relative to those dependent on participant recall or subjectivity.

Our analytic plan was designed specifically to improve estimation of the reliability of clinically relevant predictors of COPD severity and activity. Inherent variability in a predictor, due either to measurement error or to short-term biological fluctuations, may bias estimates of the association between the predictor and an outcome [16]. Estimation of the reliability of a predictor, such as the intraclass correlation, allows correction for the bias using regression calibration. This is important in defining COPD subsets for analysis and in evaluating disease progression.

These results relate to the complexity of what we call COPD. Historically, the disease has been conceptualized as having a slow, often variably progressive decline in lung function. [17–19]. However, recent data suggests that the slope of decline in FEV_1_% predicted slows in advanced disease[20] and that correlations between lung function and other measures such as exacerbations and symptoms are not always strong [21, 22]. Nevertheless, FEV_1_ remains the objective gold standard for assessing disease severity, progression, and treatment efficacy. Our finding that all PFTs had an ICC >0.90 supports the use of PFTs as a primary outcome variable. These data are consistent with other reports demonstrating the high repeatability of spirometric values between visits of short intervals over a range of disease severities [12, 23]. Our mean change in FEV_1_ (20.4 mL overall, 30.7 mL for men, 1.9 mL for women), compares favorably with results from the Lung Health Study (LHS) at screening visits 21 days apart, which reported a coefficient of variation of 4% (changes of 14.3 mL for men, 4.5 mL for women)[23]. Similar results were reported for FEV_1_ in the National Emphysema Treatment Trial (NETT) for PFTs conducted within 60 days of each other [12]. We extend those finding by showing that IC, an important driver of exercise limitation, was as repeatable as other spirometric parameters (ICC = 0.93) [24–26]. IC decreases during exercise in COPD, and is responsive to bronchodilator therapy [27, 28]. Bronchodilator response is influenced by many factors including pre-bronchodilator FEV_1_ and actual withholding of confounding bronchodilator medications [29]. In our study, withholding bronchodilators was not required, but extensive questions were asked on drug use and time of administration. Our reported bronchodilator response was calculated in subjects who withheld bronchodilators (65 out of 98 subjects) for sufficient time before PFT testing, potentially representing a more accurate picture of bronchodilator responsiveness.

Fully assessing the full clinical picture of COPD requires additional factors and composite scores dependent on patient recall and subjective evaluation [30]. We found that most of the validated questionnaires had lower ICCs than the PFTs. Among them, the most repeatable was the SGRQ-C, which is tailored for use in chronic airflow limitation and is responsive to changes in disease activity [31, 32]. Repeatability of SGRQ-C has been reported as ICC = 0.92 in 40 subjects evaluated at a 2 week interval [31], in good agreement with our results. Each questionnaire has a different specified recall time period, ranging from none specified (SGRQ-C) to 7 days for the FACIT-F and current assessment for the CAT, which could affect repeatability. Nevertheless, we found significant correlations between the utilized questionnaire scores (data not shown), suggesting that they are measuring similar aspects of the disease. This relationship is not surprising because all aim to assess the functional impact of COPD on quality of life. Our finding that repeatability of subject recall of age started smoking was similar to assessing average number of cigarettes smoked over their life-time, is consistent with an extensive internet based survey of tobacco exposure and risk [33]. They found age first started smoking cigarettes slightly more consistent (ICC = 0.85) than calculated pack-years (ICC = 0.76). Despite our finding of a few subjects with very different recall of pack-years smoked, overall, pack-years seems relatively reliable, but of unknown accuracy.

Finally, these results provide insights into the use of patient recall of exacerbation history to predict susceptibility to future exacerbations. Our results on short-term repeatability of recalling COPD exacerbations before enrollment (total exacerbations, kappa = 0.42), was somewhat higher when defined by treatment with corticosteroids or antibiotics (kappa = 0.54 and 0.58, respectively). These data are similar to reported concordance of cardiovascular events, in that the more defined the event, the greater the reliability [34]. It was surprising that recall of exacerbations requiring an emergency department visit or hospitalization was not significantly higher. The frequency of exacerbations, and not the severity, may be the most important factor in patient recall inaccuracies [35]. In the ECLIPSE study, patient recall of the number of exacerbations in the year before enrollment was the single strongest predictor of future exacerbations [22] highlighting the importance of exacerbation recall for COPD studies. However, our current results imply that using patient recall of exacerbation frequency as an enrollment criterion for short-term clinical trials may lead to substantial variability in outcomes. The inaccuracy of patient recall of exacerbation rate was recently highlighted when compared with single-physician chart review or a central adjudication committee [35]. It is surprising that recall of hospitalization or ED visit was not consistent, highlighting the need for exacerbation documentation in determining GOLD status and associated treatment choices.

A key feature of our analysis is its implications for the GOLD combined assessment of COPD, which is employed both for assessment of disease severity and for treatment recommendations. We found a higher reliability (as judged by ICC) for the GOLD classification using PFTs. Incorporation of symptom scores (MMRC and CAT) into GOLD classification for symptoms and exacerbation risk increases variability between visits. Using the 2015 GOLD combined assessment criteria, 31% of the subjects changed by at least one GOLD level using GOLD-MMRC and 18% changed using GOLD-CAT. We recognize that there are two possible interpretations of the more limited repeatability of symptom-based scores compared to PFTs. It is possible that symptom scores are more sensitive than PFTs for clinical changes and provide a more sensitive indicator of current illness. This is reflected in the updated GOLD criteria [15, 36]. Symptom scores also reflect an integrated assessment of a multi-organ disease and comorbidities that are common in these subjects. However, the observation that parameters dependent upon subject recall also have poorer consistency upon repeat evaluation suggests that a component of the variation in symptoms, in the absence of changes in spirometry, may be a reflection of the subjective nature of the assessment. Further work is required to best delineate the precision of change in symptom scores as endpoints for clinical trials in COPD.

As with all studies, there are limitations. This Repeatability Substudy evaluated differences in participant response over an interval of 28.9±9.4 days (mean ± SD). We did not adjust for variation in time between visits. It is also unknown how a longer or shorter time would correlate with the results reported here. Though demographics of the Substudy were consistent with the total SPIROMICS cohort, only 7 of the 11 clinical centers contributed data to this Substudy, so these results may not reflect repeatability across all of the centers. Also, we cannot rule out an effect of training [37], as some measures improved at the repeat visit. Perhaps most crucially, these subjects may not be representative of the general COPD population.

In summary, we demonstrate that in the SPIROMICS Repeatability Substudy, the repeatability of supervised objective measures was strongest. PFTs demonstrated the highest ICC values and recall of exacerbations had some of the lowest kappa statistics. Reliability of questionnaires was consistent with literature reports. However, significant numbers of subjects had variation between visits above recommended MCIDs, which notably affected GOLD staging for symptoms and exacerbation risk incorporating either the CAT or MMRC. Within-subject variability must be accounted for in interpreting phenotype assignments or disease progression. We feel that these data will aid the design and interpretation of longitudinal COPD studies. Examining multiple types of parameters widely used in COPD assessment should also help in the development of clinical practice guidelines.

## Supporting information

S1 Fig PFTs and bland-altman plots. Subjects (n = 96) are color coded by GOLD stratification (using PFT values only). GOLD 0 = red, GOLD 1 = green, GOLD 2 = Blue, GOLD 3 = orange and GOLD 4 = Purple. The solid red line = the mean difference between the baseline and repeat visit values, the dotted red line is ± 1 SE and the Dashed blue line is ± 1 SD. A) Post-bronchodilator FVC, B) Post-bronchodilator FVC Bland-Altman Plot, C) Post-bronchodilator FEV_1_/FVC, D) Post-bronchodilator FEV_1_/FVC Bland-Altman Plot, E) Post-bronchodilator Inspiratory Capacity and F) Post-bronchodilator Inspiratory Capacity Bland-Altman Plot.
(TIF)

S1 Study Information (DOCX)
