# Supplementary figures and images for: Variability in objective and subjective measures affects baseline values in studies of patients with COPD
Source: PLoS One. 2017 Sep 21;12(9):e0184606. doi: 10.1371/journal.pone.0184606 (PMC5608200; doi:10.1371/journal.pone.0184606)

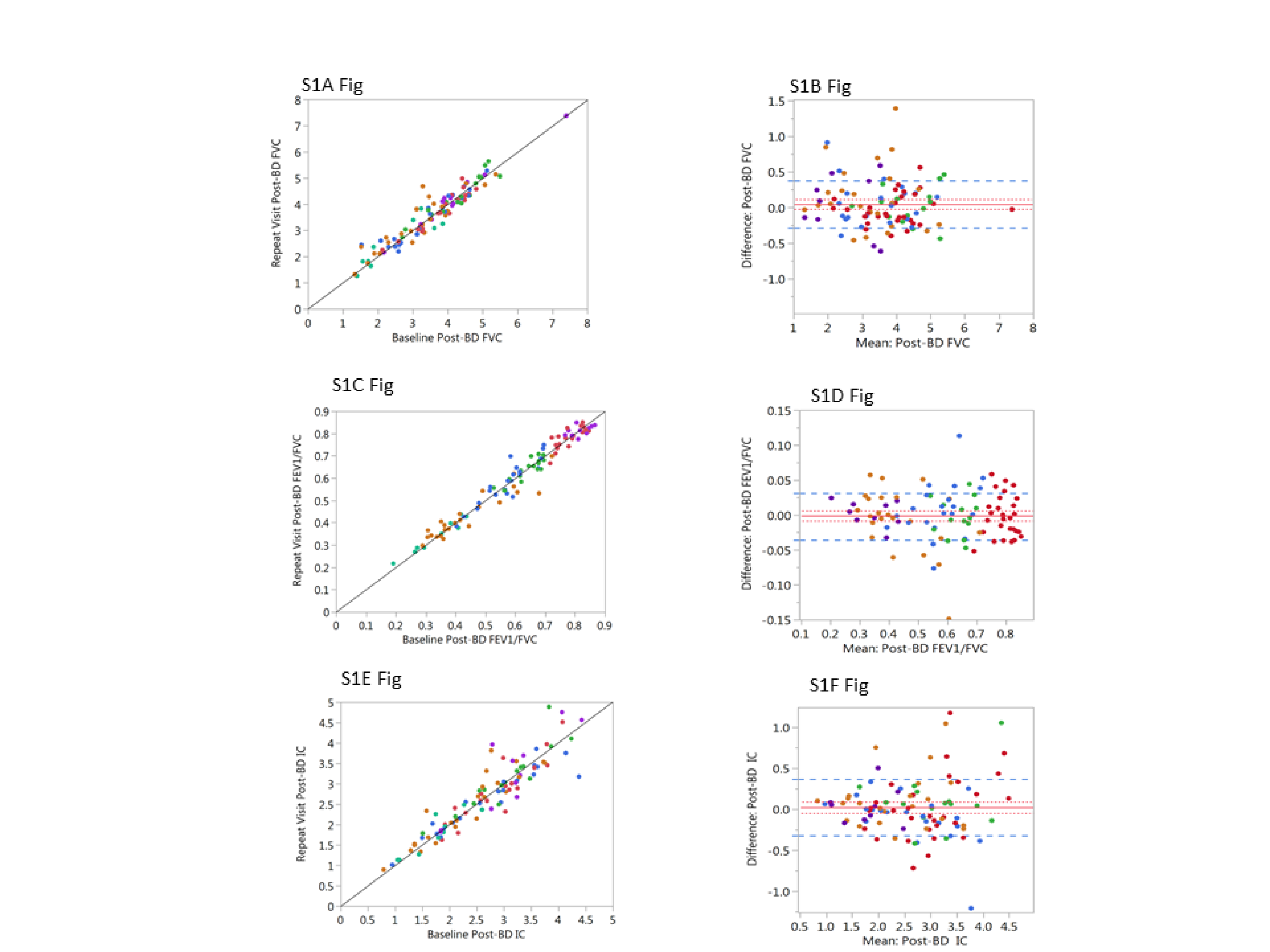

Supplement: S1 Fig — Subjects (n = 96) are color coded by GOLD stratification (using PFT values only). GOLD 0 = red, GOLD 1 = green, GOLD 2 = Blue, GOLD 3 = orange and GOLD 4 = Purple. The solid red line = the mean difference between the baseline and repeat visit values, the dotted red line is ± 1 SE and the Dashed blue line is ± 1 SD. A) Post-bronchodilator FVC, B) Post-bronchodilator FVC Bland-Altman Plot, C) Post-bronchodilator FEV1/FVC, D) Post-bronchodilator FEV1/FVC Bland-Altman Plot, E) Post-bronchodilator Inspiratory Capacity and F) Post-bronchodilator Inspiratory Capacity Bland-Altman Plot. (TIF) [file pone.0184606.s001.tif]
