# Supplementary material for: Variability in objective and subjective measures affects baseline values in studies of patients with COPD
Source: PLoS One. 2017 Sep 21;12(9):e0184606. doi: 10.1371/journal.pone.0184606 (PMC5608200; doi:10.1371/journal.pone.0184606)
Supplement: S1 Study Information — (DOCX) [file pone.0184606.s002.docx]

**S1 Supporting Information**

**Methods:**

The 2005 ATS/ERS guidelines for pulmonary function testing and interpretation served as the primary guidance for the conduct and interpretation of the PFTs measurements [[1](#_ENREF_1), [2](#_ENREF_2)] and the 2002 ATS statement guided the six-minute walk test [[3](#_ENREF_3)]. The between-maneuver repeatability, which 90% of consecutive patients can meet, is 120 ml (6.1%) for FEV1 and 150 ml (5.3%) for FVC [[4](#_ENREF_4)]. The established target is 150 ml for both measures (or 100 ml if the FVC is <1 L) [[1](#_ENREF_1)]. The short-term (24.9±17.1 days) reproducibility in mild COPD participants is 113 ml (CV 4.1%) for FEV1 and 150 ml (CV 3.5%) for FVC (10). The minimal clinically important difference for FEV1 is approximately 100 ml [[5](#_ENREF_5)]. Prior to PFTs, participants were asked to withhold/refrain from vigorous exercise (0.5 hours), smoking (1 hour), eating a large meal (2 hours), alcohol (4 hours), caffeine (6 hours), inhaled albuterol (6 hours), inhaled ipratropium (8 hours), any twice daily bronchodilators for 24 hours and any once daily bronchodilators for 48 hours.

**Study Personnel:**

Ashley Angeles, BA (University of California at San Francisco, San Francisco, CA); Andrea P Anguila (University of California at Los Angeles, Los Angeles, CA); Carlos Azucena (University of California at Los Angeles, Los Angeles, CA); Cassandra Almonte (Columbia University, New York, NY); Jessica A Baker, BS (University of Utah Hospitals and Clinics, Salt Lake City, UT); Joanna Barkas (Columbia University, New York, NY); Chad Baumgardner (National Jewish Health, Denver, CO); Amanda Beller (Collaborative Studies Coordinating Center, Chapel Hill, NC); Andrew Belli (Johns Hopkins University, Baltimore, MD); Nina Bracken, MSN, ACNP-BC (University of Illinois at Chicago, Chicago, IL); Ashley D Britt (Collaborative Studies Coordinating Center, Chapel Hill, NC); Jeanette P Brown, MD, PhD (Immunophenotyping Core, University of Michigan, Ann Arbor, MI); Michael Brown (Nspire/PTF Reading Center, University of California at Los Angeles, Los Angeles, CA); Hope Bryan (Collaborative Studies Coordinating Center, Chapel Hill, NC); Robert Buchanan (Nspire/PTF Reading Center, University of California at Los Angeles, Los Angeles, CA); Judy Carle, MPH (University of Utah Hospitals and Clinics, Salt Lake City, UT); Crystal Cannon (University of Illinois at Chicago, Chicago, IL); John J Carney, BS (University of California at Los Angeles, Los Angeles, CA); Elizabeth Carretta, MPH, PMP (Collaborative Studies Coordinating Center, Chapel Hill, NC); Cate Castleberry, BS (University of Michigan, Ann Arbor, MI); Christina Christensen (University of Iowa Carver College of Medicine, Iowa City, IA); Sean W Crudgington, MS (Immunophenotyping Core, University of Michigan, Ann Arbor, MI); Cheryl M. Clare, AA (Johns Hopkins University, Baltimore, MD); Griselda Compres (Columbia University, New York, NY); Teresa L. Concordia (Johns Hopkins University, Baltimore, MD); Joy M Cook, BS (Collaborative Studies Coordinating Center, Chapel Hill, NC); Helga Criner, RN (Temple University, Philadelphia, PA); Gang Cui (Collaborative Studies Coordinating Center, Chapel Hill, NC); Elijah Darnell, BA (University of California at San Francisco, San Francisco, CA); Hendrik Dejong (Collaborative Studies Coordinating Center, Chapel Hill, NC); Julie DeLisa (University of Illinois at Chicago, Chicago, IL); Forrest DeMarcus (Collaborative Studies Coordinating Center, Chapel Hill, NC); John C Dermand, BA (University of California at Los Angeles, Los Angeles, CA); Kyra Engelberg, MA (University of California at Los Angeles, Los Angeles, CA); Kimberly Estell, BS (University of Alabama at Birmingham, Birmingham, AL); Brian C Fedor (University of California at Los Angeles, Los Angeles, CA); Jessica Foft, BA (University of California at San Francisco, San Francisco, CA); Rebecca Forney (Johns Hopkins University, Baltimore, MD); Susan Foster, PhD (Wake Forest Medical Center, Winston-Salem, NC); Cori Fratelli, BA (National Jewish Health, Denver, CO); Stacey Fulwiler, BA (University of California at San Francisco, San Francisco, CA); Suresh Garudadri, BS (University of California at San Francisco, San Francisco, CA); Vanessa Gonzales (University of Illinois at Chicago, Chicago, IL); Arianna Gonzalez (University of California at Los Angeles, Los Angeles, CA); Rachel West Goolsby (Collaborative Studies Coordinating Center, Chapel Hill, NC); Blair Gordy, BA (Wake Forest Medical Center, Winston-Salem, NC); Natalia Gouskova (Collaborative Studies Coordinating Center, Chapel Hill, NC); Laura Grammer (Johns Hopkins University, Baltimore, MD); Victoria Groysberg (University of California at Los Angeles, Los Angeles, CA); Edwin Ha (University of California at Los Angeles, Los Angeles, CA); Jae Wook Ha (Collaborative Studies Coordinating Center, Chapel Hill, NC); Necole Harris, BS, CRT (University of Alabama at Birmingham, Birmingham, AL); Hannah Haymond (University of Utah Hospitals and Clinics, Salt Lake City, UT); Terri Herrud, BS,CCRP (National Jewish Health, Denver, CO); Robert Hinderer (University of Michigan, Ann Arbor, MI ); Bob Hmieleski, MBA (Wake Forest Medical Center, Winston-Salem, NC); Grace Ibrahim (University of California at Los Angeles, Los Angeles, CA); Michele M Inishi (Collaborative Studies Coordinating Center, Chapel Hill, NC); Michael P. Johnson (University of Utah Hospitals and Clinics, Salt Lake City, UT); Briona Jones (Johns Hopkins University, Baltimore, MD); Kyle Jung, BA (University of California at San Francisco, San Francisco, CA; Rufino Junta (University of California at Los Angeles, Los Angeles, CA); Samar K Kadi, RN (University of California at Los Angeles, Los Angeles, CA); Lynn Kelso, RPFT, RCPT (Nspire/PTF Reading Center, University of California at Los Angeles, Los Angeles, CA); Barbara Kerns, RTPFT, CRTT (Nspire/PTF Reading Center, University of California at Los Angeles, Los Angeles, CA); Kyle Killeen, MS (Columbia University, New York, NY); Christopher Kirby, MS, CPFT, CPT (University of California at San Francisco, San Francisco, CA); Jeff Krings, FNP (Wake Forest Medical Center, Winston-Salem, NC); Marisa Kwock, BA (University of California at San Francisco, San Francisco, CA); Denise Lao, BS (University of California at San Francisco, San Francisco, CA); Michael Li, BS (University of California at San Francisco, San Francisco, CA); Angelica Lopez (Columbia University, New York, NY); Dinah Lorenzo (University of California at Los Angeles, Los Angeles, CA); Barbara C MacDonald, RN (Temple University, Philadelphia, PA); Nell Malone (Collaborative Studies Coordinating Center, Chapel Hill, NC); Chenna Mandapati (Temple University, Philadelphia, PA); Michelle Maye, RRT (Nspire/PTF Reading Center) (University of California at Los Angeles, Los Angeles, CA); Cheryl Majors, RN, BA (University of Michigan, Ann Arbor, MI); Carla Martin, BS (Wake Forest Medical Center, Winston-Salem, NC); Keenya Mathews, BS (University of Alabama at Birmingham, Birmingham, AL); Hope McGehee (National Jewish Health, Denver, CO); Lizbeth McPherson, BA (University of California at San Francisco, San Francisco, CA); Heena Mehta (Collaborative Studies Coordinating Center, Chapel Hill, NC); Laura N Menck, MA, CCRC (University of California at Los Angeles, Los Angeles, CA); Argie L Mina (University of California at Los Angeles, Los Angeles, CA); Milka J Monegro, BA (Columbia University, New York, NY); Amelia Mutso, PhD (University of Illinois at Chicago, Chicago, IL); Michael Newstead (University of Michigan, Ann Arbor, MI ); Meg Nielsen (University of Utah Hospitals and Clinics, Salt Lake City, UT); Christine Nguyen, BS (University of California at San Francisco, San Francisco, CA); Cindy Nguyen, BS (University of California at San Francisco, San Francisco, CA); Audrey Novak (Johns Hopkins University, Baltimore, MD); Stefanie M. Nuez, BS (University of California at Los Angeles, Los Angeles, CA); Debra J. O’Connell-Moore, BS (University of Iowa Carver College of Medicine, Iowa City, IA); Janet S Orin, RPFT (University of California at Los Angeles, Los Angeles, CA); Milian Patel (University of California at Los Angeles, Los Angeles, CA); Amelie Peisl (Columbia University, New York, NY); Jeffrey Peng, BS (University of California at San Francisco, San Francisco, CA); Claudia L Perdomo, AS (University of California at Los Angeles, Los Angeles, CA); P.M.Quibrera MSc. (Collaborative Studies Coordinating Center, Chapel Hill, NC); Rebeca Ramirez (University of California at San Francisco, San Francisco, CA); Brian Rector, MS (Wake Forest Medical Center, Winston-Salem, NC); Christian Rodriguez (University of California at Los Angeles, Los Angeles, CA); Allison Rogalski, MS (Columbia University, New York, NY); Kathy Roggenkamp (Collaborative Studies Coordinating Center, Chapel Hill, NC); Kelly A. Rysso, BS (University of Michigan, Ann Arbor, MI); John Sage (Nspire/PTF Reading Center, University of California at Los Angeles, Los Angeles, CA); Rhonda Schissler (Nspire/PTF Reading Center, University of California at Los Angeles, Los Angeles, CA); Christina Schnell (National Jewish Health, Denver, CO); Meagan Scott (Johns Hopkins University, Baltimore, MD); Daniel D Shin (Columbia University, New York, NY); Michael Sims (Nspire/PTF Reading Center, University of California at Los Angeles, Los Angeles, CA); Shelly Smith, MS (Wake Forest Medical Center, Winston-Salem, NC); Teniola Sodeinde (Columbia University, New York, NY); Joanne Sonstein (Immunophenotyping Core, University of Michigan, Ann Arbor, MI); Penny Spernoga, MS (Wake Forest Medical Center, Winston-Salem, NC); Kim Sprenger (University of Iowa Carver College of Medicine, Iowa City, IA); Marilyn Stahle (Nspire/PTF Reading Center, University of California at Los Angeles, Los Angeles, CA); Michelle Staudt (Columbia University, New York, NY); Lisa M Stearns, CCRT (University of California at Los Angeles, Los Angeles, CA); Valerie R Stolberg, MPH (Immunophenotyping Core, University of Michigan, Ann Arbor, MI); Brenzell Summers, BS (Wake Forest Medical Center, Winston-Salem, NC); Summer Sun (Collaborative Studies Coordinating Center, Chapel Hill, NC); Taylor Thorn, BS (National Jewish Health, Denver, CO); Jennifer Underwood (National Jewish Health, Denver, CO); Jason T Varasteh (National Jewish Health, Denver, CO); Michelle Vig (Nspire/PTF Reading Center, University of California at Los Angeles, Los Angeles, CA); Martin Villegas (University of Utah Hospitals and Clinics, Salt Lake City, UT); Marietta Wadley, RCP (University of California at Los Angeles, Los Angeles, CA); Jingming Wang, BS (University of California at San Francisco, San Francisco, CA); Timothy Wang, BS (University of California at San Francisco, San Francisco, CA); Jillian Welker (National Jewish Health, Denver, CO); Heather Wells, BS (Collaborative Studies Coordinating Center, Chapel Hill, NC); John Wheeler (University of California at Los Angeles, Los Angeles, CA); Harold Winnike (University of Iowa Carver College of Medicine, Iowa City, IA); Kelsey Wollen, BA (University of California at San Francisco, San Francisco, CA); Jeffery R Wood, MBA (Nspire/PTF Reading Center, University of California at Los Angeles, Los Angeles, CA); Sundos Yassin, BA (University of California at San Francisco, San Francisco, CA); Imamah Younus, BA (University of California at Los Angeles, Los Angeles, CA); Jennifer Zielger (University of Iowa Carver College of Medicine, Iowa City, IA); Liujian Zhao (University of Michigan, Ann Arbor, MI )

**Supporting Information Captions**

**S1 Fig: PFTs and Bland-Altman Plots.** Subjects (n=96) are color coded by GOLD stratification (using PFT values only). GOLD 0=red, GOLD 1= green, GOLD 2= Blue, GOLD 3=orange and GOLD 4= Purple. The solid red line = the mean difference between the baseline and repeat visit values, the dotted red line is ± 1 SE and the Dashed blue line is ± 1 SD. A) Post-bronchodilator FVC, B) Post-bronchodilator FVC Bland-Altman Plot, C) Post-bronchodilator FEV_1_/FVC, D) Post-bronchodilator FEV_1_/FVC Bland-Altman Plot, E) Post-bronchodilator Inspiratory Capacity and F) Post-bronchodilator Inspiratory Capacity Bland-Altman Plot.

**Supporting Information References**

1. Macintyre N, Crapo RO, Viegi G, Johnson DC, van der Grinten CP, Brusasco V, et al. Standardisation of the single-breath determination of carbon monoxide uptake in the lung. The European respiratory journal. 2005 Oct;26(4):720-35. PubMed PMID: 16204605. Epub 2005/10/06. eng.

2. Miller MR, Hankinson J, Brusasco V, Burgos F, Casaburi R, Coates A, et al. Standardisation of spirometry. The European respiratory journal. 2005 Aug;26(2):319-38. PubMed PMID: 16055882. Epub 2005/08/02. eng.

3. ATS statement: guidelines for the six-minute walk test. American journal of respiratory and critical care medicine. 2002 Jul 1;166(1):111-7. PubMed PMID: 12091180. Epub 2002/07/02. eng.

4. Enright PL, Beck KC, Sherrill DL. Repeatability of spirometry in 18,000 adult patients. American journal of respiratory and critical care medicine. 2004 Jan 15;169(2):235-8. PubMed PMID: 14604836. Epub 2003/11/08. eng.

5. Donohue JF. Therapeutic responses in asthma and COPD. Bronchodilators. Chest. 2004 Aug;126(2 Suppl):125S-37S; discussion 59S-61S. PubMed PMID: 15302773. Epub 2004/08/11. eng.
